# Ear-ache

**Published:** 1875-09

**Authors:** 


### EAR-ACHE.

Parents and all having the care of infants, should watch carefully for this very painful complaint. The pain from inflammation and tumefaction of the membranes of the ear are intense, and the complaints attending are generally attributed to colic or anything but the true cause. Some pernicious and poisonous "soothing" stuff is given, and the child ceases to cry because its senses are benumbed. Sometimes when the pain appears to be in the ear the nurse thursts a bit of scalding hot onion or fig into it, and so increases the torture.

A child's ears should be washed daily with warm water, and very thoroughly. But the operation should be gently performed as the interior parts are very sensitive and may be easily injured. If any discharge is discovered, let a little warm water be dropped in from a sponge, and retained for a couple of minutes, and then poured out and again repeated. The practice of rolling up the corner of a napkin and crowding it down into the ear to clear it, is a dangerous one, as it forces more of the secretions down upon the ear-drum than it removes. Faithful daily soaking with warm water, the water being poured out by turning the child upon its side, is the true cleansing process. If pain is believed to exist in the region of the ear, warm flannels often re-warmed, will often cause relief. In severe cases, anodynes may be externally employed with safety. Warm fomentations of soaked poppy leaves often harmlessly narcotize the parts. Ear-ache of teething children is often caused from sympathy with the parts involved in the process of dentition. In such cases cloths wrung out in very warm, though not scalding water, and applied to the jaws and neck, have a soothing effect.

In short, moderate heat and moisture, however applied, will be found the great sedative. Bear in mind that it is infinitely better that a child should not be soothed and quieted, than that it should be narcotized by alcohol or opium or soothing syrups or mixtures of anise-seed or teas of mint, all of which are very bad for the feeble brain, and any one of which is capable of destroying infant life, if given copiously or in small doses for a long time. Better that the child suffer now, than weaken his hold on life or beget in him a desire for stimulants which will endure forever.

---

London, as a city of odors, is not attractive. There are in the city fifty courts and alleys, with 536 houses, in which the intermittent water supply now furnished is received either in ruinous old water-butts, often in the cellars, and exposed to emanations from sewers or closets, or in cisterns open to every kind of accidental or designed pollution. In one such cistern, the report tells us, "boys have been seen to wash their feet;" and the inhabitants of some of the houses are compelled to store water in open vessels in their often crowded living and sleeping rooms, where in a very short time it becomes loaded with organic impurities and living organisms. Cleanliness is impossible and disease is rife. Some of the details are too disgusting for repetition, but they are the inevitable results of the water which has been described. Many of these courts are inhabited by foreign Jews, who are not naturally cleanly in their habits, but who in London could not be cleanly if they would; and Dr. Saunders protests "against the cowardice and injustice of blaming for their dirty habits people who are compelled to use the same scanty measure of water times over for washing or for cooking purposes, and who, if they are thirsty, must go to a public-house, because their filthy water-butts and cisterns yield only what is undrinkable, and not enough even of that."

---

Save your suds for the garden and plants, or to harden yards when sandy.

